# Stenting of bifurcation lesions

**DOI:** 10.1007/s12471-017-0973-4

**Published:** 2017-03-06

**Authors:** N. H. J. Pijls, S. J. L. Leus, F. M. Zimmermann, L. X. van Nunen, M. van’t Veer, J. Koolen, E. van Hagen

**Affiliations:** Department of Cardiology, Catharina Hospital Eindhoven, Eindhoven, The Netherlands

To the Editor,

We appreciate the positive comments of Dr Stella and Dr Wykrzykowska [1] on our paper on bifurcation stenting in the Catharina Hospital, Eindhoven [2] and would like to react as follows:

1. RCT versus our retrospective analysis.

We agree that an RCT cannot be compared with a retrospective analysis. However, we do not agree on the note of natural selection bias. On the contrary, we believe that our analysis is completely unbiased because literally all patients admitted for bifurcation stenting in the year 2013 were included.

In a randomised controlled trial with strict inclusion and exclusion criteria, the population is more selected.

2. Baseline characteristics.

The baseline characteristics as presented in Table 1 of our paper refer to patient characteristics. The angiographic characteristics are mentioned separately and of course these are different between the two groups. That is also what we emphasise in our paper and in the discussion we suggest that the angiographic complexity might be one of the reasons to grade up from a provisional stent strategy to a more complex stent strategy.

3. Medina classification.

We choose for the Medina classification because this is the most commonly used classification and most well-known to general interventionalists.

4. Clinical restenosis rates.

We do not agree that the MACE rates at one year are relatively high. As presented in Table 3 of our paper, the mortality was 5.5% and 7.3% in the two groups at one year. Thanks to the ‘Meetbaar Beter’ project, which is a joint registry for all Dutch heart centres, [3] we know that in patients with a comparable age the 1‑year mortality for all PCIs in that age group for all Dutch heart centres with surgical back-up (including Maastricht UMC and AMC Amsterdam) ranges from 4 to 7%. Therefore, when taking into account that bifurcation stenting will be at least not less complex than the average PCI and considering that in 44 patients an LM/LAD/LCX bifurcation was present, these mortality data are exactly in line with the averages of all Dutch heart centres. A similar note can be made for MI rates and TVR rates.

This does not take away the fact that a bifurcation PCI is a ‘different animal’ as stated by Stella and Wykrzykowska. And we agree completely about what they call the KISS approach (keep it safe and simple). That was the bottom line of our paper and our message would be: ‘Start simple, grade up if mandatory’.
